# Binding Affinity of a Highly Sensitive Au/Ag/Au/Chitosan-Graphene Oxide Sensor Based on Direct Detection of Pb^2+^ and Hg^2+^ Ions

**DOI:** 10.3390/s17102277

**Published:** 2017-10-06

**Authors:** Nur Hasiba Kamaruddin, Ahmad Ashrif A. Bakar, Nadhratun Naiim Mobarak, Mohd Saiful Dzulkefly Zan, Norhana Arsad

**Affiliations:** 1 Department of Electric, Electronic and Systems Engineering, Faculty of Engineering and Built Environment, Universiti Kebangsaan Malaysia, 43600 UKM Bangi, Selangor, Malaysia; hasiba@siswa.ukm.edu.my (N.H.K.); saifuldzul@ukm.edu.my (M.S.D.Z.); noa@ukm.edu.my (N.A.); 2 School of Chemical Sciences and Food Technology, Faculty of Science and Technology, Universiti Kebangsaan Malaysia, 43600 UKM Bangi, Selangor, Malaysia; nadhratunnaiim@ukm.edu.my

**Keywords:** binding affinity, chitosan–graphene oxide, multi-metallic, lead, mercury

## Abstract

The study of binding affinity is essential in surface plasmon resonance (SPR) sensing because it allows researchers to quantify the affinity between the analyte and immobilised ligands of an SPR sensor. In this study, we demonstrate the derivation of the binding affinity constant, K, for Pb^2+^ and Hg^2+^ ions according to their SPR response using a gold/silver/gold/chitosan–graphene oxide (Au/Ag/Au/CS–GO) sensor for the concentration range of 0.1–5 ppm. The higher affinity of Pb^2+^ to binding with the CS–GO sensor explains the outstanding sensitivity of 2.05 °ppm^−1^ against 1.66 °ppm^−1^ of Hg^2+^. The maximum signal-to-noise ratio (SNR) upon detection of Pb^2+^ is 1.53, and exceeds the suggested logical criterion of an SNR. The Au/Ag/Au/CS–GO SPR sensor also exhibits excellent repeatability in Pb^2+^ due to the strong bond between its functional groups and this cation. The adsorption data of Pb^2+^ and Hg^2+^ on the CS–GO sensor fits well with the Langmuir isotherm model where the affinity constant, K, of Pb^2+^ and Hg^2+^ ions is computed. The affinity of Pb^2+^ ions to the Au/Ag/Au/CS–GO sensor is significantly higher than that of Hg^2+^ based on the value of K, 7 × 10^5^ M^−1^ and 4 × 10^5^ M^−1^, respectively. The higher shift in SPR angles due to Pb^2+^ and Hg^2+^ compared to Cr^3+^, Cu^2+^ and Zn^2+^ ions also reveals the greater affinity of the CS–GO SPR sensor to them, thus supporting the rationale for obtaining K for these two heavy metals. This study provides a better understanding on the sensing performance of such sensors in detecting heavy metal ions.

## 1. Introduction

The development of a surface plasmon resonance (SPR) sensor in various applications such as drug discovery, disease monitoring and effluent detection normally originates in the possible interactions between the analyte of interest and its immobilised ligands on the surface of the sensing layer [1]. Macromolecular interactions which result in successful binding between the analyte and ligands will vary the refractive index in the vicinity of the sensing surface, thus manipulating the response of the SPR sensor. The binding affinity constant, K, is a parameter that quantifies the effectiveness of these macromolecular interactions [2].

The binding affinity constant provides quantitative information on the binding interactions between the analyte and sensing layer, which explains the sensing performance of an SPR sensor in terms of sensitivity and selectivity. Evaluating the binding affinity under interference of a multiple-analyte system can provide an information on the selectivity of the sensor [3]. Binding interaction under various conditions, such as different pH values, enables the determination of the right regeneration mechanism of an SPR sensor [4]. Analysing the binding affinity at varying temperatures can also extract the thermodynamic nature of the macromolecular interactions [5].

The derivation of the binding affinity constant is normally based on the kinetic and thermodynamic model [6]. Nevertheless, the SPR technique provides a practical tool for retrieving such a constant by leveraging the Langmuir isotherm model, which is analogous to the kinetic approach [7]. However, careful interpretation is needed such that the derived value can be regarded as the “apparent binding affinity constant” instead of at equilibrium. This is to accommodate the kinetic controls on the macromolecular interactions that might still be occurring between the analyte and ligands during measurement. The practicality of this technique in deriving the value of K inspired many researchers to study the binding affinity of various types of analyte, including heavy metal ions on different SPR sensors [8,9,10,11].

Heavy metals refer to any metallic element with atomic density four to five times greater than water, such as lead (Pb), mercury (Hg), arsenic (As) and cadmium (Cd) [12]. Assuming that atomic density and toxicity are interrelated, heavy metals are considered detrimental to the environment and organisms [13]. Therefore, their detection plays an important role in monitoring the emission of effluents in our environment. The most common techniques for detecting heavy metals are mass spectroscopy, atomic absorption spectrometry [14], inductively coupled plasma mass spectrometry (ICPMS) [15,16] and microwave induced plasma atomic emission spectroscopy (MIP–AES) [17,18]. Although these detection techniques are known for their high sensitivity, the technologies involve expensive instrumentation that is complex to operate and immobile. Therefore, electrochemical techniques such as fluorimetry [19], colorimetry [20] and voltammetry [21,22,23] have been studied to provide a cheaper alternative offering simplicity and mobility.

Anodic stripping voltammetry (ASV) is one of the commonly used electrochemical techniques that can achieve a very low limit of detection (LOD), i.e., in ppt levels. For instance, Lu et al. fabricated an ASV glassy carbon electrode (GCE) coated with graphene, Au nanoparticles and CS that can detect down to 1 ppt of Pb^2+^ ions [24]. Although this technique is appealing for its low LOD and portability through miniaturisation, the interference from several species in the analyte can degrade the sensitivity of the electrode. On the other hand, Zhou et al. leveraged the ion interference to simultaneously detect Pb^2+^ and Cd^2+^ ions using L-cysteine/graphene–chitosan GCE [25]. Even though simultaneous detection is possible through multiplexing circuitry, an individual electrode still needs to be fabricated and optimised for each analyte, hence limits to the practicality of this approach.

Other than the electrochemical techniques, an SPR sensor is another attractive method that also offers cost-efficiency and simplicity. Although many SPR sensors for heavy metal detection have been developed, their portability has hardly been examined. Nevertheless, recent work by several researchers has shown that the portability of this sensor is viable through fabrication with optical fibre [26,27]. Not only that, some SPR sensors based on smartphone platforms have also been studied for the same purpose [28,29]. Progressive interest in the portability of SPR sensing technology shows a promising future for this sensor for in situ heavy metal detection. Consequently, various types of SPR sensor for heavy metal detection have been employed in order to further explore the potential of this device.

Recently, Wang et al. presented a study on the competitive adsorption of albumin for the detection of Cu^2+^ ions and other heavy metal ions in tap water [10]. The study exhibited an explicit analysis of the SPR data using the Langmuir isotherm model to obtain the dissociation constant, K_d_. Considering the definition of K_d_ as the reciprocal of K [7], the values of binding affinity deduced from this study are relatively small, i.e., 2.4 × 10^2^ and 4.3 × 10^2^ for both Pb^2+^ and Hg^2+^, in comparison with the values obtained by other SPR sensors in Table 1. The small values of K obtained by Wang and his co-workers suggests that albumin might not be a good ligand for heavy metal ions.

The magnitude of the binding affinity depends on the strength of the interactions between the heavy metal ions and immobilised ligands of the SPR sensor. Chitosan (CS) is known for its high affinity towards heavy metal ions such as Pb^2+^ and Hg^2+^ due to abundant amino groups (–NH_2_) that bind easily with such cations [33]. Therefore, CS has been widely used as the sensing layer of SPR sensors for heavy metal detection [30,34,35]. McIlwee et al. studied the performance of CS as the SPR sensor for Fe^3+^ ions, and proposed 9.49 × 10^5^ M^−1^ to describe quantitatively the binding affinity between the cation and CS. Abdi et al. added polypyrrole to the CS nanocomposite to improve its sensitivity to Pb^2+^ and Hg^2+^ ions [34]. The binding affinity of the polypyrrole–CS was further analysed using Cu^2+^, Zn^2+^ and Ni^2+^ ions, which yielded the binding affinity constants of 1.3 × 10^4^, 2.3 × 10^4^ and 1.7 × 10^4^, respectively [31,32].

Other materials that have recently driven many studies in SPR sensing are graphene-based materials such as graphene oxide (GO), which has a large surface area and high π-conjugation structure that significantly improve the sensitivity of an SPR sensor [36,37,38]. In addition, GO supports the surface plasmon in the visible range, and its planar sheet structure provides extra protection to the SPR sensor [36]. Apart from that, GO also carries a significant volume of hydroxyl (−OH), carbonyl (C=O) and carboxylic (C(=O)OH) functional groups, which are readily available binding sites for the heavy metal ions [39].

The abundance of functional groups in CS and GO that are readily available for binding with the heavy metal ions has prompted the development of a CS–GO nanocomposite as an SPR sensor. The first CS–GO SPR sensor, which was fabricated by Lokman et al., demonstrated an outstanding sensitivity of 1.1 °ppm^−1^ for 5 ppm Pb^2+^ ions. However, the conventional CS–GO SPR sensor on a single Au layer has a limited linearity range of only up to 1 ppm [40]. Therefore, an advanced CS–GO SPR sensor based on an Au/Ag/Au multi-metallic nanostructure was proposed to provide an enhanced evanescent field which can further detect the increasing concentration of Pb^2+^ ions without saturation, thus extending the existing linearity range [41]. However, previous studies on the Au/CS–GO and Au/Ag/Au/CS–GO SPR sensors did not include work to derive the binding affinity constant.

Therefore, the aim of this study is to quantify the binding affinity of Pb^2+^ and Hg^2+^ to an Au/Ag/Au/CS–GO SPR sensor by deriving their binding affinity constant, K. It is important to highlight that the Au/Ag/Au/CS–GO SPR sensor was used in this study to ensure a strong and stable evanescent field is maintained in order to detect the whole range of ion concentration, i.e., 0.1 ppm, 0.5 ppm, 1 ppm, 3 ppm and 5 ppm, without saturation. The extended linearity range due to the enhanced evanescent field is important for providing sufficient SPR data for the whole range of heavy metal concentrations, thus enabling the binding affinity constants to be derived accurately. Both cations were considered in this study due to their strict maximum contaminant levels (MCL) in drinking water of 0.015 ppm and 0.002 ppm, as per established by the United States Environmental Protection Agency (US EPA). However, since the Au/Ag/Au/CS–GO SPR sensor was proposed for heavy metal detection in groundwater, the range of concentration was based on the toxicity characteristic leaching procedure (TCLP), which is the lab procedure that sets the regulatory level of leachable effluent such as Pb^2+^ and Hg^2+^ that might leach into soil and groundwater and hence be used as drinking water. According to the US EPA, the threshold levels for Pb^2+^ and Hg^2+^ which were determined by this procedure were 5 ppm and 0.2 ppm, respectively [42]. The Langmuir isotherm model was used to derive the binding affinity constants for both heavy metal ions and the results were compared. This study is essential for validating the performance of such a sensor in detecting the emission of these heavy metal ions in our environment.

To the best of our knowledge, this is the first derivation of K for Pb^2+^ and Hg^2+^ ions on a CS–GO SPR sensor that quantitatively describes the interaction between them, thereby confirming the superior performance of such a sensor in heavy metal monitoring. The sensing performances were presented in terms of sensitivity, linearity range, repeatability and signal-to-noise ratio (SNR).

## 2. Materials and Methods

### 2.1. Fabrication of Au/Ag/Au/CS–GO SPR Sensor

**Figure 1:**
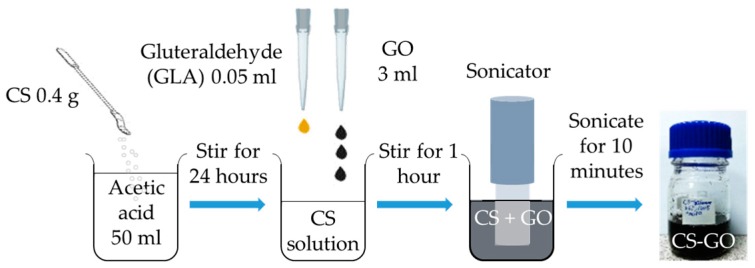
The in-house synthesis of the CS–GO nanocomposite.

The CS–GO nanocomposite was prepared in-house by dispersing 0.4 g chitosan (Sigma Aldrich, St. Louis, MO, USA) into 50 mL of 1% acetic acid and stirring this for 24 h. The cross-linking of CS was achieved by adding 0.05 mL glutaraldehyde (GLA), and then the synthesis of the CS–GO nanocomposite was continued by combining 3 mL of GO into the solution. The nanocomposite was stirred for another hour, then the process finally proceeded to a 10 min sonication to achieve homogeneity. Figure 1 illustrates the synthesis of the CS–GO nanocomposite.

**Figure 2:**
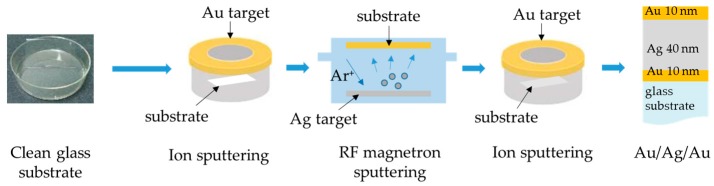
Deposition of multi-metallic nanostructures.

The multi-metallic Au/Ag/Au nanostructures were coated on a glass substrate by initially sputtering a 10 nm bottommost Au layer. The subsequent 40 nm Ag inner layer was deposited using a K.J. Lesker PVD 75 RF magnetron sputtering machine in an argon atmosphere. The working pressure and RF power were set to 0.67 Pa and 50 W, respectively. The 10 nm topmost Au layer was finally deposited to cover the Ag layer. Both Au depositions were performed in a vacuum using a Hitachi E-1010 ion sputtering machine. The working pressure and discharge current were set to 10 Pa and 15 mA, respectively. All depositions were carried out at room temperature using targets of 99.99% purity. Figure 2 depicts the deposition process in making the multi-metallic Au/Ag/Au nanostructures.

The final step in the fabrication method is immobilising the CS–GO ligands by spin-coating 1 mL of the nanocomposite on to the multi-metallic nanostructures at 6000 rpm rotational speeds for 30 s. One sample was fabricated for each concentration of both Pb^2+^ and Hg^2+^ ion solutions.

### 2.2. Characterisation of Au/Ag/Au/CS–GO Nanostructure

In this study, an energy dispersive X-ray (EDX) analysis was performed to obtain the elemental composition of the Au/Ag/Au/CS–GO SPR sensor using a Zeiss/SUPRA 55VP Field Emission Scanning Electron Microscope (FESEM) operated at 15 kV. Additionally, sectional analysis and the height profile of the CS–GO nanolayer were also obtained using an NT-MDT NtegraPrima Atomic Force Microscope (AFM).

The cross section of the Au/Ag/Au/CS–GO SPR sensor has been discussed in a previous publication, together with other characteristics such as surface topography, crystallinity, molecular composition and chemical bonding [41].

### 2.3. Execution of SPR Measurement

**Figure 3:**
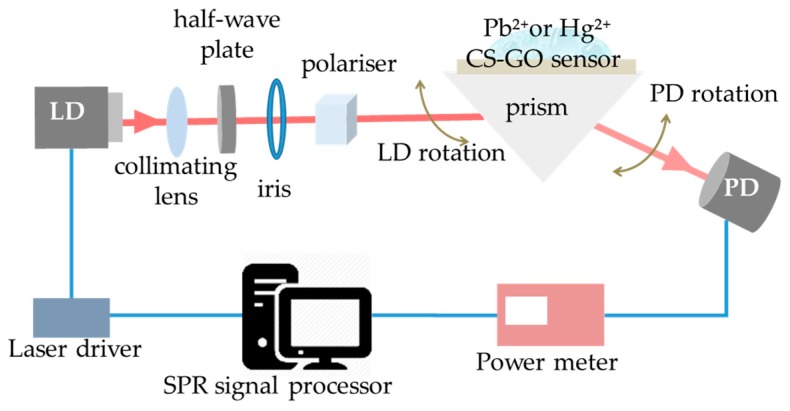
SPR spectroscopy based on the Kretschmann configuration.

The SPR measurement in this study was realised using lab-scale SPR spectroscopy based on the Kretschmann configuration, as in Figure 3. In this set-up, an 850 nm excitation light source was provided by a single mode vertical cavity semiconductor-emitting laser diode (LD). A collimating lens was used to minimise any divergence in the excitation source, thus producing an output beam of parallel rays. In order to control the amount of light passing through the system, an iris was mounted together with the collimator. Finally, the light travelled through a half-wave plate and a polarising beam splitter, where it was manipulated to illuminate only the p-polarised light.

The p-polarised light, which is the prerequisite for exciting the SPR, was then given angular incidence to the BK 7 prism. The angular modulation of the incident light was set from 20–90°, where the reflected beam was detected by a photodetector (PD) and measured using a power meter. These procedures, i.e., p-polarised light angular modulation, reflected power retrieval and data manipulation, were executed by the SPR signal processor, which was then developed in the LabVIEW environment in order to plot the SPR reflectivity curves.

The automation steps of the SPR signal processor were performed after the dispersion of analyte on to the Au/Ag/Au/CS–GO SPR sensor. These procedures were repeated five times for each Pb^2+^ and Hg^2+^ ion concentration. The analytes were prepared from the standard solutions (1000 ppm) of Pb^2+^ and Hg^2+^ ions. Five different concentrations i.e., 0.1 ppm, 0.5 ppm, 1 ppm, 3 ppm and 5 ppm were diluted from each cation standard solution using deionised water (DIW). The DIW was of resistivity 18 megohm-cm in order to minimise potential interference from other species in the analyte during measurement.

## 3. Results and Discussion

### 3.1. Elemental Composition of Au/Ag/Au/CS–GO SPR Sensor

Table 2 shows the elemental composition of the CS–GO sensing surface obtained using the EDX. The analysis shows the elements detected on the surface of the sensor, i.e., carbon (C), oxygen (O), Ag and Au. Notably, O is more dominant than other elements of greater content in the CS–GO SPR sensor. This is because the CS that was grafted on to the GO promotes the presence of O on the surface of the sensor. Since the superficial nature of EDX only detects the prominent element on the surface, O prevails over other elements in this analysis [43]. The effects of grafting and chemical modification also suppressed the detection of nitrogen (N) in CS, despite its higher volume than GO [44]. Similarly, the weight percentage of N in the CS–GO nanocomposite that was synthesized by Kumar and Jiang was also the lowest compared to other elements [45]. Furthermore, the weight percentage of Ag and Au were significantly high, which could also be one of the reasons for the undetected N in this analysis. On a positive note, the weight percentage of Ag and Au proves the availability of the multi-metallic nanostructures in the CS–GO SPR sensor.

### 3.2. Cross-Sectional Analysis and Height Profile of the CS–GO Nanolayer

**Figure 4:**
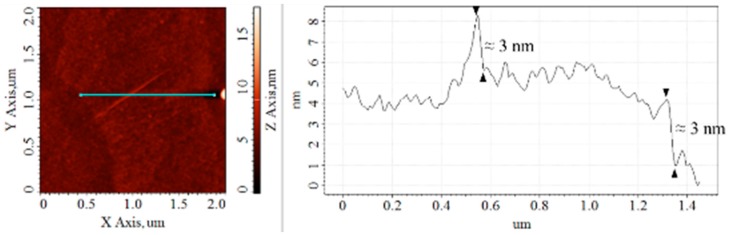
The cross-sectional analysis that computes the thickness of the CS–GO nanolayer.

The cross-sectional analysis of the CS–GO nanolayer is indicated by the blue line in the left image of Figure 4, while the plot on the right is the height profile produced by it. The estimated thickness of the CS–GO nanolayer based on the vertical displacement, which is marked by the arrows in the height profile, is about 3 nm, while that measured by the FESEM in our previous study was about 5 nm [41].

The thickness of GO depends on its degree of exfoliation, where complete exfoliation down to an individual sheet may yield a minimum thickness of approximately 0.8 nm. The functionalisation of CS on to the GO sheets has notably changed the latter structure, hence the increase in its thickness. The thickness of CS–GO nanocomposites obtained by Bustos-Ramirez, Kim, Yan and their co-workers are in the range of 3–6 nm, 3–4 nm and 3 nm, respectively [43,46,47]. Clearly, the thickness of the CS–GO nanolayer in this study is in accordance with these CS–GO nanocomposites, which infers that the CS has been successfully functionalised on to the GO layer through our in-house synthesis.

### 3.3. SPR Reflectivity in Pb^2+^ and Hg^2+^ Ions

**Figure 5:**
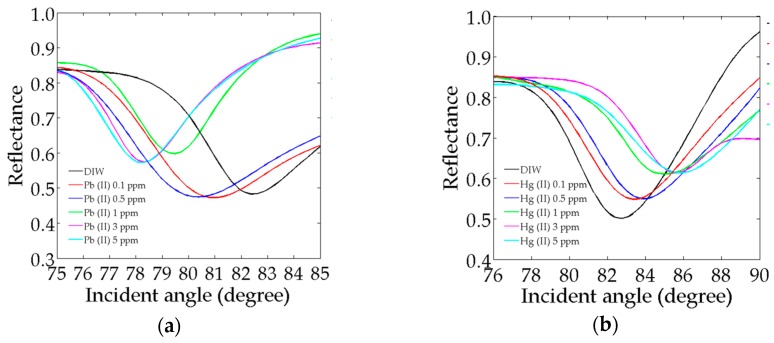
SPR reflectivity of the Au/Ag/Au/CS–GO SPR sensor in (**a**) Pb^2+^ and (**b**) Hg^2+^ ion.

Figure 5 exhibits the SPR reflectivity curves of the Au/Ag/Au/CS–GO SPR sensor upon adsorption of Pb^2+^ and Hg^2+^ ion concentrations. In Figure 5a, the Au/Ag/Au/CS–GO SPR sensor depicts a negative-shift from the reference SPR angle, which is that of the deionised water (DIW), i.e., 82.5°. The SPR angles for 0.1–5 ppm Pb^2+^ ions move towards smaller values, which are approximately 81.0°, 80.3°, 79.5°, 78.3° and 78.0°. The positive-shift of the SPR angle is more frequently found in the literature compared to the negative-shift. This is because most SPR sensors produce an increase in the refractive index of the sensing layer due to the analyte-ligand interaction. However, there are several other SPR sensors that exhibit negative-shift behavior for various reasons [40,48,49,50,51]. According to Saha and Sarkar, the adsorption of As (III) ions on to their CS-based SPR sensor might have reduced the thickness of the sensing layer, which consequently decreased the SPR angle with the cation concentration [50]. On the other hand, Singh and Gupta proposed that the negative-shift of their SPR sensor was due to the decrease in refractive index as a result of a chemical reaction between glucose and oxygen at the sensing layer [51]. Lokman et al. [40] as well as Fen and his co-workers [48,49] did not justify the negative-shift produced by their SPR sensors. Therefore, it is inferred that the Pb^2+^ adsorption on to the CS–GO sensing matrix might have caused other effects that are not only limited to the increase in refractive index and thus yielded the negative-shift.

A rather classic reasoning attributes this phenomenon to the leaky waves, which propagate in a backward direction at the metal-dielectric interface [52,53]. The thickness of the Au/Ag/Au/CS–GO SPR sensor, which exceeds 60 nm [54], and the multi-metallic nanostructures, which are capable of exciting multiple SPR [55], could also be reasons for the uncommon SPR response. Nonetheless, the results are deemed acceptable as the direction of the shifts are consistent for all measurements with Pb^2+^ ions, and this behavior is known a priori.

On the contrary, Figure 5b shows the SPR reflectivity curves upon adsorption of Hg^2+^ ions exhibiting a positive-shift. It shows that the SPR angle moves to greater values as the concentration of Hg^2+^ increases, which contradicts the results obtained for Pb^2+^ ions. This is a manifestation of several effects. First, the adsorption of the Hg^2+^ ions on to the CS–GO matrix might have increased the latter’s refractive index, thus producing the positive-shift in the SPR angle. Second, the binding event between the Hg^2+^ ions and CS–GO nanolayer might have not caused any conformational changes to the latter, hence the effective thickness of the sensor remains unaffected, thus maintaining the positive-shift for the whole range of concentration [50].

#### Full-Width-Half-Maximum (FWHM)

Moving on, the full-width-half-maximum (FWHM) of the SPR curves for the Au/Ag/Au/CS–GO SPR sensor in Pb^2+^ are roughly 5.5° and 5.0° for 0.1 ppm and 0.5 ppm; meanwhile, the higher region of concentration, i.e., 1–5 ppm, demonstrates a smaller FWHM of approximately 3.0°. The fact that the FWHM for each SPR curve is high might be due to the intensified internal loss produced by the increase in total thickness of Au, as per previous studies [56,57]. The fact that smaller FWHM is observed for the higher concentration of Pb^2+^ ions could be due to the increased binding event with the available functional groups of the CS–GO nanolayer, which reduced the scattering of free electrons and consequently narrowed the FHWM [58]. Although a smaller FWHM, e.g., less than 1°, would be more appealing for a better detection accuracy, this result is still acceptable as it accords with the FWHM obtained for the Au/Ag/Au SPRi chip, i.e., 3° [59].

Next, the FWHM of the SPR curves in Hg^2+^ ions are about 6.0°, 5.5°, 5.0°, 4.5° and 4.5°. The FWHM produced by each Hg^2+^ ion concentration is generally higher by 1.0° than those due to Pb^2+^ ions. Since there is limited literature discussing the variation of FWHM by various heavy metal ions, a direct comparison with other studies seems unachievable. However, the broadening of the SPR curves in Hg^2+^ could be due to greater surface plasmon scattering produced by that cation compared to Pb^2+^ [60,61].

### 3.4. Calibration Curves for Pb^2+^ and Hg^2+^ Ions

**Figure 6:**
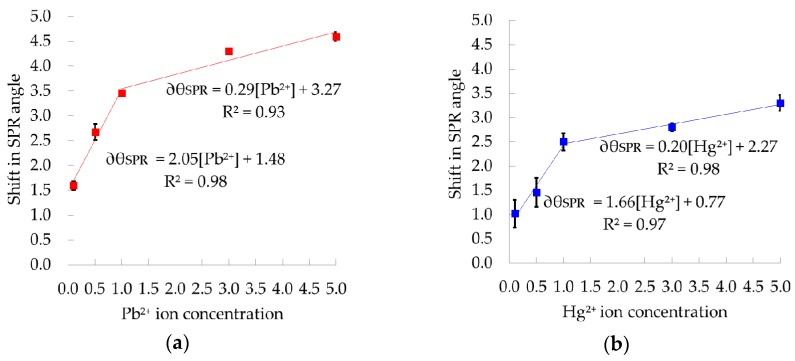
Calibration curve of the Au/Ag/Au/CS–GO SPR sensor in (**a**) Pb^2+^ and (**b**) Hg^2+^ ion.

Figure 6a,b shows the calibration curves computed from the shift of the SPR angle. The shifts of the SPR angle for each Pb^2+^ concentration of 0.1, 0.5, 1, 3 and 5 are approximately 1.6°, 2.7°, 3.5°, 4.3° and 4.6°, in the same order. ∂θ_SPR_ is linearly dependent on [Pb^2+^] in two regions of concentration, i.e., 0.1 ppm to 1 ppm and 1 ppm to 5 ppm. The calibration curves for both regions of Pb^2+^ concentration are given by the following equations, with correlation coefficients R^2^ of 0.98 and 0.93, respectively.

∂θ_SPR_ = 2.05[Pb^2+^] + 1.48  0.1 ppm ≤ [Pb^2+^] ≤ 1 ppm (1)

∂θ_SPR_ = 0.29[Pb^2+^] + 3.27  1 ppm ≤ [Pb^2+^] ≤ 5 ppm (2)

As shown in Figure 6b, the shifts in the SPR angle for 0.1, 0.5, 1, 3 and 5 ppm of Hg^2+^ are about 1°, 1.5°, 2.5°, 2.8° and 3.3°, respectively. Similar trends are also demonstrated by the SPR response of the Au/Ag/Au/CS–GO SPR sensor in Hg^2+^ ions, which is described by the equations below. The linear regression analysis yielded correlation coefficients R^2^ of 0.97 and 0.98 for the lower and higher concentration regions, respectively.

∂θ_SPR_ = 1.66[Hg^2+^] + 0.77  0.1 ppm ≤ [Pb^2+^] ≤ 1 ppm (3)

∂θ_SPR_ = 0.20[Hg^2+^] + 2.27  1 ppm ≤ [Pb^2+^] ≤ 5 ppm (4)

The equations show that the ∂θ_R_ is directly correlated to the concentration of both cations. The CS–GO SPR sensor also shows an excellent linearity range with no sign of saturation for both cations. This is due to the enhanced Ag inner layer, which provides a stronger evanescent field to enable further detection of heavy metal ions as compared with the CS–GO SPR sensor on a single Au layer [40].

The gradient of the curves signify the sensitivity of the CS–GO sensor within that range of concentration. The Au/Ag/Au/CS–GO SPR sensor is more sensitive towards Pb^2+^ than Hg^2+^ in both regions of concentration based on the higher gradient values. The sensitivity in the lower and higher concentration regions for Pb^2+^ are 2.05 °ppm^−1^ and 0.29 °ppm^−1^, respectively. Meanwhile, the sensitivity towards Hg^2+^ is 1.66 °ppm^−1^ and 0.20 °ppm^−1^ in the same order.

Similar results were also observed where a polypyrrole–chitosan thin film exhibits greater sensitivity to Pb^2+^ than Hg^2+^ [34]. The higher sensitivity of the CS–GO SPR sensors towards Pb^2+^ is clearly due to its high affinity towards this ion as compared to Hg^2+^. The Irving–Williams series for affinity to most organic ligands also follows the same order, i.e., Pb^2+^ > Hg^2+^. The Pb^2+^ ionic radii of 1.21 Å, which is bigger than that of Hg^2+^ (1.10 Å), was also claimed to be one of the reasons for the stronger affinity towards the CS–GO binding sites [62].

#### Repeatability

The repeatability of the Au/Ag/Au/CS–GO SPR sensor towards Pb^2+^ and Hg^2+^ are quantified by the relative standard deviation (RSD) of the average shift in SPR angle based on five repetitions. This performance parameter is represented by the error bars in the calibration curve. It shows clearly that the magnitude of error bars are greater for Hg^2+^, which indicates that more variation had occurred during its detection than for Pb^2+^. Therefore, the Au/Ag/Au/CS–GO SPR sensor is less repeatable when tested with Hg^2+^ than Pb^2+^.

The repeatability can be related to the stability of the bond that was formed between these heavy metal ions and the CS–GO nanolayer. The Pearson’s acid-base concept suggested that soft acids react faster and bind stronger with soft bases. A similar concept is also applicable between hard acids and hard bases [63]. Certain heavy metals such as Cu^+^, Ag+, Cd^2+^ and Hg^2+^ are categorised as soft acids that bind faster and stronger with ligands containing sulfur. Meanwhile, borderline acids like Ni^2+^, Cu^2+^, Zn^2+^ and Pb^2+^ are more likely to bind with nitrogen donor atoms [64,65]. The CS–GO that is loaded with the amine groups (–NH_2_) might have formed a stronger bond with Pb^2+^ compared to Hg^2+^, hence less variation has occurred during the detection of the former cation, thus yielding a lower RSD. As per the result, higher repeatability was obtained for Pb^2+^ than Hg^2+^ ions [66,67].

### 3.5. Binding Affinity Constant, K

**Figure 7:**
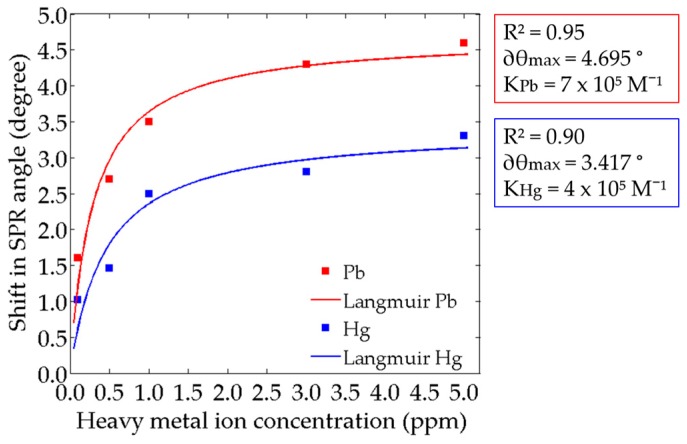
Langmuir isotherm model of the SPR angle shift for Pb^2+^ and Hg^2+^ ions.

In order to model the binding affinity of the Pb^2+^ and Hg^2+^ ions towards the CS–GO sensing layer, the calibration curves were fitted to the analogous Langmuir isotherm model, which is expressed as below:

(5) $$\partial_{SPR}=\;\frac{\partial \theta_{\max}\;\left[M^{2+}\right]}{\;\frac{1}{K}+\left[M^{2+}\right]}$$

where ∂θ_max_ is the maximum SPR shift or the SPR shift at saturation, $\left[M^{2+}\right]$ is the concentration of the heavy metal, and K is the affinity constant. The plots, which are fitted in the Langmuir model though the MATLAB fitting tool, are exhibited in Figure 7.

The fitting of the calibration curve for Pb^2+^ using the Langmuir model isotherm model yielded an R^2^ value of 0.95 and ∂θ_max_ of approximately 4.695°, which is about 0.095° higher than the experimental maximum shift of the SPR angle, i.e., 4.6°. Similar analysis was also performed on the calibration curve for Hg^2+^ where a slightly lower value of R^2^ was obtained, i.e., 0.90. The value of ∂θ_max_ for Hg^2+^ ions is about 3.417°, which is also greater than the experimental value of 3.3°. The fitting outcome shows that the calibration curve for Pb^2+^ fits better to the Langmuir isotherm model compared to that for Hg^2+^.

From this model, the binding affinity constants, K, for Pb^2+^ and Hg^2+^ to the Au/Ag/Au/CS–GO were also derived. It is calculated that the value of K for Pb^2+^ is about 7 × 10^5^ M^−1^ while 4 × 10^5^ M^−1^ for Hg^2+^. A higher affinity constant is observed for Pb^2+^, which infers that the CS–GO layer is more attracted to Pb^2+^ than Hg^2+^. This could be due to the higher electronegativity of Pb^2+^ than Hg^2+^, which accounts for a stronger interaction with the CS–GO layer. Following the Pauling scale of electronegativity, the Pb^2+^ has a value of 2.33 over 2.00 of Hg^2+^ [68]. Most importantly, the values of K for Pb^2+^ and Hg^2+^ in this study are comparable with those obtained using the listed SPR sensors in Table 1.

However, it is crucial to note that the derivation of the binding affinity constants via SPR response is rather challenging because it is based on the variation in the refractive index in the vicinity of the CS–GO layer due to the changing concentration of Pb^2+^ or Hg^2+^. Thus, it may not represent the overall kinetic behavior of the binding. It is important, therefore, to note that the estimated binding affinity constant using this model is an “apparent affinity”, since the reaction may still be under kinetic control and have yet to reach equilibrium [8,9,10,11]. However, as suggested by previous authors, the analogous Langmuir isotherm model should be seen as a practical tool that facilitates the determination of reasonable kinetic parameters for a better understanding of the binding event between the analyte and ligands [7].

Other than that, it is also worth highlighting that the multi-metallic nanostructure has indeed facilitated the derivation of the binding affinity constant by extending the linearity range, thus providing sufficient data for fitting the Langmuir isotherm model.

### 3.6. Detection Accuracy and Signal-to-Noise-Ratio

**Figure 8:**
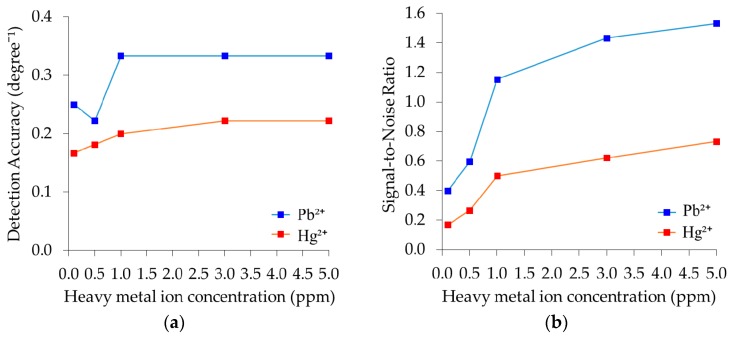
(**a**) Detection accuracy and (**b**) Signal-to-noise ratio of the Au/Ag/Au/CS–GO SPR sensor in Pb^2+^ and Hg^2+^ ions.

Detection accuracy (DA) is the inverse of the FWHM which measures how accurately the SPR angle was obtained from the SPR curve. It depends solely on the width of the SPR curve. The narrower the FWHM given, the higher the DA. Figure 8a shows the DA of the Au/Ag/Au/CS–GO SPR sensor upon adsorption of Pb^2+^ and Hg^2+^. It can be observed that the DA for 0.1 and 0.5 ppm of Pb^2+^ ions are approximately 0.25/° and 0.22/°, where the value for 1–5 ppm is 0.33/°. On the other hand, the DA in Hg^2+^ increased with concentration, i.e., from about 0.17/°, 0.18/°, 0.20/°, and remains stagnant at 0.22/° for 3 ppm and 5 ppm.

On average, the DA of the Au/Ag/Au/CS–GO SPR in Hg^2+^ is roughly 0.10/° lower than the values in Pb^2+^. This result signifies that the FWHM of the sensor is higher in Hg^2+^ than when it was exposed to Pb^2+^. Apart from the probability that Hg^2+^ might have induced greater surface plasmon scattering due to its weaker bond with CS–GO, some shortcomings during the measurements could also produce undesired noise that broadens the FWHM [69].

Another performance parameter that combines the effects of ∂θ_SPR_ and DA is the signal-to-noise ratio (SNR). The SNR can be regarded as the basic figure-of-merit (FOM) of an SPR sensor and is determined by multiplying ∂θ_SPR_ by DA. The SNR of the Au/Ag/Au/CS–GO SPR sensor in both heavy metal ions is depicted in Figure 8b. It is notable that despite the ambiguous variation of DA, the SNR for each CS–GO SPR sensor still increases with the Pb^2+^ concentration. Such a result shows that SNR is also another indication of binding affinity [3] because the quantity is highly dependent on the ∂θ_SPR_. This also demonstrates that the shape of the SNR curve mirrors the shift in the SPR angle, which shows that ∂θ_SPR_ has greater effect than DA in determining the value of SNR.

Notably, the SNR is higher in Pb^2+^ than Hg^2+^ for each concentration i.e., 0.40, 0.60, 1.15, 1.43 and 1.53 for 0.1 ppm, 0.5 ppm, 1 ppm, 3 ppm and 5 ppm, respectively. Following the same order, the SNR for each concentration of Hg^2+^ is 0.17, 0.27, 0.5, 0.7 and 0.66, respectively. The SNR of Pb^2+^ for each concentration is more than twice that of Hg^2+^, which is due to the higher affinity of CS–GO towards the former than the latter. Sharma et al. suggested that a logical criterion of an SNR for bi-metallic SPR fiber optic SPR sensors should be around 0.6 and above [70]. The values of SNR for Pb^2+^ in this study are definitely higher than the proposed value, thus supporting the reliability of the results.

### 3.7. Shift in SPR Angle for Various Heavy Metal Ions

**Figure 9:**
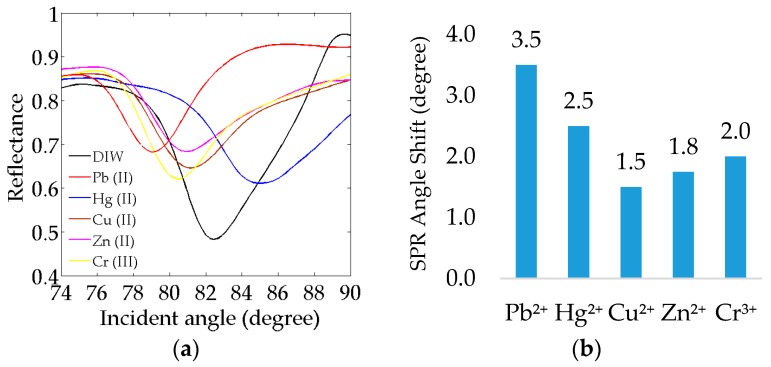
(**a**) SPR reflectivity and (**b**) Shift in SPR angle of the Au/Ag/Au/CS–GO SPR sensor in 1 ppm of Pb^2+^, Hg^2+^, Cu^2+^, Zn^2+^ and Cr^3+^.

Figure 9a exhibits the SPR curves of the Au/Ag/Au/CS–GO SPR sensor in 1 ppm of Pb^2+^, Hg^2+^, Cu^2+^, Zn^2+^ and Cr^3+^. The SPR angles for 1 ppm of Cu^2+^, Zn^2+^ and Cr^3+^ are roughly 81.8°, 81.4° and 80.8°, respectively. The SPR curves of 1 ppm Pb^2+^ and Hg^2+^ were also plotted in the same graph for comparison. Apparently, all analytes produced a negative-shift towards the lower values of the SPR angle from the reference (82.5°) except Hg^2+^. The similar trend between Pb^2+^, Cu^2+^ and Zn^2+^ is due to their similar chemistry as borderline acids [63]. Because of this, their binding event with the CS–GO nanolayer might have decreased the refractive index of the latter, thus yielding a negative-shift. A similar trend was also observed for the trivalent ion Cr^3+^.

Conversely, the only SPR curve which shifts towards the higher values of the SPR angle is that of Hg^2+^. The reason for such variation is because Hg^2+^ is the only ion that falls into the category of soft acids which might interact differently with the CS–GO nanolayer. The binding between Hg^2+^ and the CS–GO nanolayer has increased the latter’s refractive index, which is translated into the positive-shift.

The shift in SPR angle for each heavy metal ion was also measured in order to further study the affinity of the Au/Ag/Au/CS–GO SPR sensor. Figure 9b depicts the shift of the SPR angle for Pb^2+^ (3.5°) prevailing over Hg^2+^, Cr^3+^, Zn^2+^ and Cu^2+^ of 2.5°, 2.0°, 1.8° and 1.5°, respectively. Based on the graph, it is shown that the CS–GO matrix has a different extent of affinity towards each cation, which in turn affects the amount of adsorption that causes the change in the refractive index, and hence the shift in SPR angle. The different extent of affinity between the heavy metal ions and CS–GO SPR sensors could be due to their individual value on the Pauling scale of electronegativity, i.e., 1.90 for Cu^2+^, 1.65 for Zn^2+^ and 1.66 for Cr^3+^, which might have caused them to interact uniquely with the –NH_2_, –OH and –COOH functional groups of the CS–GO matrix [68]. Apparently, Pb^2+^ and Hg^2+^, with higher values of electronegativity, i.e., 2.33 and 2.00, respectively, have produced greater shifts in SPR angle, signifying that the CS–GO has a higher affinity towards them compared with the other heavy metal ions. This result also supports the rationale for deriving the binding affinity of these two cations instead of the others.

On a different note, it is worth highlighting that the SPR response that is solely based on the change in refractive index could not provide conclusive information in terms of which ion would cause the variation when it comes to a multiple-ions analyte. This is due to the nature of SPR sensing itself, which is a label-free technique where the change of refractive index could be induced by any ion [10,71]. Furthermore, there is no specific ligand for each heavy metal ion, and henceforth developing a selective SPR sensor for this analyte can be very challenging [72]. Due to this consideration, it is more favourable to highlight herein the study of the binding affinity of the Au/Ag/Au/CS–GO SPR sensor than selectivity. Nevertheless, according to Homola, the selectivity of an SPR sensor can still be interpreted by comparing the shift in SPR angle produced by several types of analyte. An SPR sensor is considered to be selective towards a target analyte if it is able to produce the highest shift in SPR angle upon exposure to that particular analyte [73].

In a nutshell, this study demonstrates the derivation of the binding affinity constant, based on the Langmuir isotherm model, in order to validate the outstanding sensitivity, repeatability, DA as well as SNR of the Au/Ag/Au/CS–GO SPR sensor in detecting Pb^2+^ ions. The extended linearity range provided by the enhanced electromagnetic field of Au/Ag/Au nanostructures facilitates the analysis by providing sufficient SPR data for the whole range of heavy metal concentration, thus enabling the binding affinity constants to be derived. This study has expanded the other sensing modality of the Au/Ag/Au/CS–GO SPR sensor for environmental monitoring.

## 4. Conclusions

An Au/Ag/Au/CS–GO SPR sensor, which is very sensitive to Pb^2+^ and Hg^2+^ ions, was deployed to realise a quantitative study on the binding affinity of these cations. The binding affinity constant, K, based on the Langmuir isotherm model, gives a value of 7 × 10^5^ M^−1^ for Pb^2+^ and 4 × 10^5^ M^−1^ for Hg^2+^. The higher value of K for Pb^2+^ signifies that the CS–GO sensing layer is more favorable to this cation than Hg^2+^, due to its greater electronegativity and ionic radii. The high binding affinity between Pb^2+^ and the multi-metallic CS–GO SPR sensor justifies the maximum sensitivity of 2.05 °ppm^−1^ as compared to 1.66 °ppm^−1^ upon exposure of Hg^2+^. The repeatability for Pb^2+^ also prevails over Hg^2+^, with 50% lower relative standard deviation (RSD) than the latter. The result also exhibits greater SNR for Pb^2+^ of 1.53, which exceeds the proposed logical criterion of an SNR, i.e., 0.6. The CS–GO SPR sensor also shows high affinity towards Pb^2+^ followed by Hg^2+^ compared to Cr^3+^, Zn^2+^ and Cu^2+^ ions. For the first time, this study has demonstrated derivation of the binding affinity parameters for Pb^2+^ and Hg^2+^ by direct detection using an Au/Ag/Au/CS–GO SPR sensor that are comparable to other SPR techniques. This finding validates the outstanding performance of the multi-metallic CS–GO SPR sensor, suggesting that its application as a heavy metal detector in environmental monitoring is promising.

## Figures and Tables

**Table 1:** Langmuir affinity binding constant, K, for various heavy metal ions using the SPR technique.

Heavy Metal Ions	SPR Sensor	K (M^−1^)	References
Ni^2+^	MUA-(His)_6_^-^	4.0 × 10^8^	[9]
Cu^2+^	MUA/Gly-Gly-His	6.0 × 10^8^	
Cu^2+^	Cysteamine/His-Gly-Gly	4.0 × 10^6^	
Fe^3+^	CS	9.5 × 10^5^	[30]
Cd^2+^	Apo-metallothionein	4.2 × 10^5^	[11]
Hg^2+^	Apo-metallothionein	2.7 × 10^3^	
Cu^2+^	Polypyrrole–CS	1.3 × 10^4^	[31]
Zn^2+^	Polypyrrole–CS	2.3 × 10^4^	[32]
Ni^2+^	Polypyrrole–CS	1.7 × 10^4^	
Cu^2+^	Albumin	2.3 × 10^2^	[10]
Pb^2+^	Albumin	2.4 × 10^2^	
Hg^2+^	Albumin	4.3 × 10^2^	

**Table 2:** The weight percentage of elements on the surface of the CS–GO nanolayer.

Element	Weight Percentage (%)
Carbon (C)	19.25
Oxygen (O)	32.62
Silver (Ag)	27.58
Gold (Au)	20.54
